# Prevalence and Determinants of Fast Food Consumption Among Medical Sciences Students in Kermanshah, Iran

**DOI:** 10.1155/sci5/2960393

**Published:** 2026-01-29

**Authors:** Maryam Janatolmakan, Shahab Rezaeian, Ali Soroush, Mahnaz Ghowsi, Alireza Khatony

**Affiliations:** ^1^ Social Development and Health Promotion Research Center, Health Policy and Promotion Institute, Kermanshah University of Medical Sciences, Kermanshah, Iran, kums.ac.ir; ^2^ Nursing and Midwifery Care Research Center, Tehran University of Medical Sciences, Tehran, Iran, tums.ac.ir; ^3^ Infectious Diseases Research Centre, Health Policy and Promotion Institute, Kermanshah University of Medical Sciences, Kermanshah, Iran, kums.ac.ir

**Keywords:** fast food, medical sciences students, prevalence, reasons

## Abstract

Recent evidence highlights a concerning increase in fast food consumption among university students. This study aimed to assess the prevalence and determinants of fast food consumption among medical sciences students in Kermanshah, Iran. A cross‐sectional study was conducted involving 300 students selected through the stratified random sampling technique. Data were collected using a structured questionnaire and a personal information form. Statistical analysis was performed using SPSS Version 18, applying both descriptive and inferential statistics, including an adjusted Poisson regression model to identify factors associated with fast food consumption. The findings showed that the prevalence of fast food consumption in the past week was 55.7%. Significant determinants included age (incidence rate ratio [IRR] = 1.51, 95% confidence interval [CI] = 1.09–2.09, and *p* = 0.012) and education level (IRR = 0.85, 95% CI = 0.75–0.97, and *p* = 0.018). No significant associations were found between fast food consumption and gender, marital status, or body mass index. The high prevalence of fast food consumption among these students raises important health concerns, reflecting unhealthy eating behaviors. Therefore, educating students about the benefits and risks of fast food consumption is essential. Moreover, improving access to healthy and nutritious food options is crucial to reduce dependence on fast food.

## 1. Introduction

Fast food refers to food that is quickly prepared and consumed, either as a full meal or a snack [1, 2]. It typically includes items such as sandwiches, sausages, pizzas, and cheeseburgers [3]. Its popularity has grown rapidly worldwide, particularly among adolescents and young adults, and it has become a symbol of unhealthy lifestyle choices [4]. Numerous studies have documented the adverse health effects of fast food consumption, including an increased risk of chronic conditions such as type 2 diabetes, colorectal cancer, fatty liver disease, metabolic syndrome, and dyslipidemia [5–8]. In particular, fast food intake is strongly associated with obesity—a major risk factor for cardiovascular disease [9]—and has even been linked to suicide attempts [9, 10].

Among university students, fast food consumption is especially prevalent [11]. Research indicates that over 50% of the students consume fast food daily [12]. Multiple factors contribute to this behavior, including affordability, availability, taste, convenience, persuasive advertising, and limited nutritional awareness [4, 13, 14].

Studies conducted in various countries have examined the prevalence and determinants of fast food consumption among students. For example, a 2023 study in Turkey reported that 40% of the students had consumed fast food in the past two weeks, primarily due to taste preferences and social settings. Similar research from Bangladesh and Saudi Arabia identified age, gender, socioeconomic status, and body mass index (BMI) as significant correlates [1, 10, 11]. Likewise, studies from Iran have reported a high prevalence of fast food intake among university students, with commonly consumed items including sandwiches, pizza, and French fries [15–17]. These studies also found associations with variables such as gender, marital status, income, and field of study. A 2021 systematic review identified common predictors of fast food consumption among students, including male gender, younger age, higher socioeconomic status, being overweight, and external factors such as taste, availability, price, and marketing strategies [14].

Studies across different countries have explored the prevalence and determinants of fast food consumption among students. For example, a 2023 study in Turkey found that 40% of the students had consumed fast food in the past two weeks, primarily due to taste and social settings. This study, along with others in Bangladesh and Saudi Arabia, identified age, gender, economic status, and BMI as significant correlates [1, 4, 18]. Similarly, studies from Iran have reported a high prevalence of fast food intake among university students, with commonly consumed items including sandwiches, pizza, and French fries [15–17]. These studies also highlighted associations with variables such as gender, marital status, income, and field of study. A 2021 systematic review identified common predictors of fast food consumption among students, including male gender, younger age, higher socioeconomic status, being overweight, and external factors like taste, availability, price, and promotions [14].

Despite extensive research, few studies have focused specifically on medical sciences students—a population expected to have higher health literacy and greater nutritional awareness. However, existing studies in Iran show inconsistent results, underscoring the need for more targeted investigations [15–17].

Therefore, the present study aims to assess the prevalence of fast food consumption and its associated factors among medical sciences students. The objectives of the study are as follows:

• To determine the prevalence of fast food consumption in the past week among medical sciences students.
• To identify factors associated with fast food consumption in this population.

## 2. Methods

### 2.1. Study Design

This cross‐sectional descriptive‐analytical study was conducted in accordance with the guidelines outlined in the Strengthening the Reporting of Observational Studies in Epidemiology (STROBE) statement [19].

### 2.2. Sample and Sampling Method

Kermanshah University of Medical Sciences (KUMS) comprises seven faculties: Nursing and Midwifery, Medicine, Pharmacy, Food Industry, Health, Dentistry, and Paramedicine. The study population included students enrolled at KUMS. Based on the findings of Mohammadbeigi et al. [17], and assuming a prevalence rate of 0.44 for fast food consumption in the past week, with a 95% confidence level, 80% power, and a precision of 0.06, the required sample size was estimated to be 263 students. The sample size was calculated using the formula: *n* = *Z* ^2^ × *p*(1 − *p*)/*d* ^2^, where *Z* = 1.96, *p* = 0.44, and *d* = 0.06. To account for a 10% nonresponse rate, the final sample size was increased to 300 students.

A stratified random sampling method was employed, with each faculty considered a stratum to ensure proportional representation across the university. Within each faculty, participants were randomly selected from updated enrollment lists using a computer‐generated random number table.

The final sample of 300 students was proportionally distributed across the faculties as follows: 60 from Paramedicine, 26 from Health, 6 from Food Industry, 52 from Nursing and Midwifery, 35 from Dentistry, 86 from Medicine, and 35 from Pharmacy. Data collection was conducted between December 2020 and February 2022.

Inclusion criteria required participants to provide informed consent. Exclusion criteria included pregnancy (due to its effects on BMI and eating habits), adherence to a special therapeutic diet, or a diagnosed chronic metabolic disorder such as diabetes mellitus, hypothyroidism, hyperthyroidism, or Cushing’s syndrome.

### 2.3. Study Instrument

Two main instruments were used in this study: a personal information form and a researcher‐developed questionnaire assessing eating habits over the past month. The personal information form gathered data on age, gender, weight, height, marital status, and education level.

The eating habits questionnaire was developed based on previous studies by AlTamimi et al. [1], Al‐Otaibi and Basuny [20], and Musaiger et al. [21]. The first section included eight multiple‐choice questions on the frequency and types of fast food consumed in the past week, such as pizza, sandwiches, falafel, cheeseburgers, chips, Caesar salad, and fried chicken. Consumption frequency was categorized as “Never,” “1–2 times,” or “≥ 3 times” during the past week.

The second section comprised 15 yes/no items to explore reasons for fast food consumption. Sample statements included the following: “I have limited time to prepare meals,” “Fast food is typically more affordable than homemade or restaurant food,” “I consume fast food primarily due to living in a dormitory or boarding house,” and “The taste of fast food is often more enjoyable than that of homemade or restaurant meals.”

To assess the questionnaire’s validity, both qualitative and quantitative content analyses were conducted. In the qualitative phase, 12 faculty members reviewed the questionnaire for relevance, simplicity, and clarity, and their feedback was incorporated. In the quantitative phase, the Content Validity Index (CVI) and Content Validity Ratio (CVR) were calculated, yielding values of 0.79 and 0.77, respectively.

Reliability was evaluated using the test–retest method. Thirty students completed the questionnaire twice with a 2‐week interval between administrations. The correlation coefficient between pretest and post‐test scores was acceptable (*r* = 0.77). These students were not part of the final study sample.

### 2.4. Data Collection

The sample size was allocated proportionally according to student enrollment in each faculty. The researcher approached students during breaks to explain the study’s objectives and obtain informed consent. Then, self‐administered questionnaires were distributed to the participants, who completed them individually and returned them to the researcher immediately after completion. Filling out the questionnaire took approximately 20 min.

### 2.5. Data Analysis

Data were analyzed using SPSS Version 16. Descriptive statistics, including means (standard deviations) and frequencies (percentages), were calculated. The primary outcome variable was the frequency of fast food consumption during the past week.

For inferential analysis, an adjusted Poisson regression model was applied to examine associations between sociodemographic variables and fast food consumption frequency. This model was preferred over alternatives, such as ordinal logistic regression, because the outcome variable (categorized as never, 1–2 times, and ≥ 3 times) represents count data and approximates a Poisson distribution. Additionally, Poisson regression provides incidence rate ratios, which facilitate interpretation in public health contexts.

The assumption of overdispersion was evaluated by comparing residual deviance to degrees of freedom; no significant overdispersion was detected, supporting the use of the standard Poisson model. Covariates included gender, age, education level, marital status, and BMI.

Although education level was presented categorically in the descriptive statistics, it was treated as a continuous variable in the regression analysis to assess the trend across increasing levels of education. Statistical significance was set at *p* < 0.05.

### 2.6. Use of AI‐Assisted Technologies

During the preparation of this manuscript, the authors used ChatGPT (OpenAI, San Francisco, CA, USA) to improve the clarity and readability of the text. After using this tool, the authors thoroughly reviewed and edited the content as needed and take full responsibility for the final version of the manuscript.

### 2.7. Ethical Considerations

This study was approved by the Ethics Committee (code: IR.KUMS.REC.1398.819). Written informed consent was obtained from all participants, and confidentiality of their information was ensured.

## 3. Results

The response rate for this study was 100%. The mean age of participants was 29.0 ± 7.5 years. The majority were female (52.0%), married (38.0%), and undergraduates (46.7%). Regarding BMI, 33.7% of the participants were classified as overweight or obese. The mean BMI for fast food consumers and nonconsumers was 23.5 ± 0.3 and 24.5 ± 3.4, respectively. The BMI range and mode for fast food consumers were 17–34 and 24, respectively; for nonconsumers, these values were 19.2–35.4 and 23.

**Figure 1:**
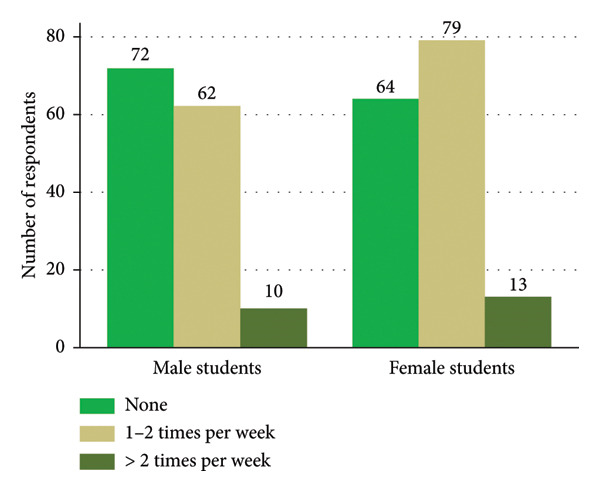
Frequency of fast food consumption by gender.

**Figure 2:**
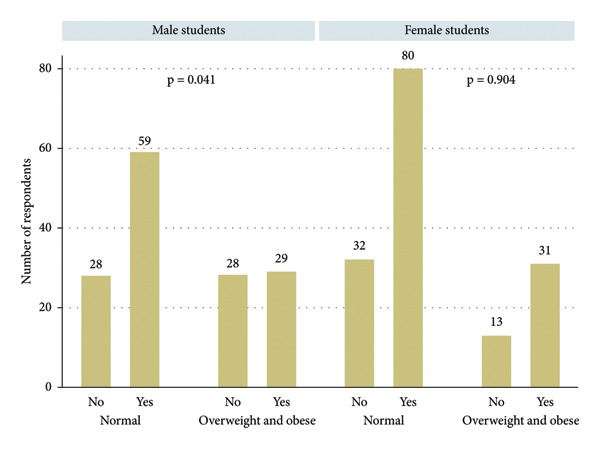
Frequency of fast food consumption by body mass index and gender.

The prevalence of fast food consumption during the past week was 55.7%. Among the participants, 53.9% of the students aged ≤ 25 years, 56.1% of the female students, 43.9% of the undergraduate students, 63.1% of the married students, and 30.5% of the overweight/obese students reported consuming fast food 1‐2 times in the past week (Table 1 and Figures 1 and 2).

**Table 1:** Demographic characteristics of students based on the frequency of fast food consumption (*N* = 300).

Variables		*n* (%)	Frequency of fast food consumption in the past week		
			Never, *n* = 136	1–2, *n* = 141	≥ 3, *n* = 23
Gender	Male	144 (48.0)	72 (52.9)	62 (43.9)	10 (43.5)
	Female	156 (52.0)	64 (47.1)	79 (56.1)	13 (56.5)

Age (year)	≤ 25	139 (46.3)	48 (35.3)	76 (53.9)	15 (65.2)
	≥ 26	161 (53.7)	88 (64.7)	65 (46.1)	8 (34.8)

Marital status	Single	186 (62.0)	81 (59.6)	89 (63.1)	16 (69.6)
	Married	114 (38.0)	55 (40.4)	52 (36.9)	7 (30.4)

Education level	BSc^†^	140 (46.7)	59 (43.4)	62 (44.1)	19 (82.6)
	MSc^‡^	61 (20.3)	21 (15.4)	37 (26.2)	3 (13.0)
	PhD^††^	33 (11.0)	17 (12.5)	15 (10.6)	1 (4.4)
	Resident	66 (22.0)	39 (28.7)	27 (19.1)	0 (0.0)

School	Paramedical	60 (20.0)	18 (13.0)	32 (22.6)	10 (43.6)
	Health	26 (8.7)	13 (10.1)	12 (8.5)	1 (4.3)
	Food industry	6 (2.0)	2 (1.5)	4 (2.7)	0 (0.0)
	Nursing and midwifery	52 (17.3)	28 (20.4)	23 (16.3)	1 (4.3)
	Dentistry	35 (11.6)	20 (14.6)	11 (7.7)	4 (17.4)
	Medicine	86 (28.7)	36 (26.4)	44 (31.2)	6 (26.1)
	Pharmacy	35 (11.7)	19 (14.0)	15 (11.0)	1 (4.3)

Body mass index (BMI)	Underweight (< 18.5)	10 (3.3)	3 (2.2)	6 (4.3)	1 (4.3)
	Normal (18.5–24.9)	189 (63.0)	84 (61.8)	92 (65.2)	13 (56.6)
	Overweight and obese (≥ 25)	101 (33.7)	49 (36.0)	43 (30.5)	9 (39.1)

^†^Bachelor of Science.

^‡^Master of Science.

^††^Doctor of Philosophy.

The most commonly consumed fast foods were pizza (78.3%), falafel (41.6%), and fried chicken (33.2%) (Table 2). The main reasons for fast food consumption were ease of access (60.0%), lack of time (50.3%), and taste (44.7%) (Table 3).

**Table 2:** Frequency of fast food consumption among students in the past week (*N* = 300).

Type of fast food	*n* (%)
Pizza	159 (78.3)
Sandwich	90 (46.6)
Falafel^∗^	108 (53.5)
Cheeseburger	46 (23.0)
Chips	60 (29.7)
Caesar salad	45 (22.4)
Fried chicken	90 (44.6)
Other	15 (7.6)

^∗^Falafel is a plant‐based food made from chickpeas and special spices that are mixed together and fried in oil.

**Table 3:** Reasons for fast food consumption among students (*N* = 300).

Items	*n* (%)
Accessing fast food is more convenient compared to home‐cooked foods.	180 (60.0)
I take pleasure in consuming fast food outdoors.	151 (50.3)
I have limited time to prepare meals.	134 (44.7)
The taste of fast food is often perceived to be more enjoyable compared with homemade and restaurant food.	127 (42.3)
I am left with no alternative but to consume fast foods.	103 (34.3)
Fast food is typically more affordable than homemade food or restaurant meals.	99 (33.0)
Fast food consumption is prevalent within my family.	91 (30.3)
I consume fast food primarily due to living in a dormitory or boarding house.	87 (29.0)
I favor fast foods due to the limited range of options available in student self‐service meals.	85 (28.3)
I opt for fast food due to the perceived low quality of student self‐service meals.	84 (28.0)
The packaging of fast foods is visually appealing.	80 (26.7)
I have a preference for high‐fat foods such as fast food.	67 (22.3)
I do not have a preference for home‐cooked foods.	38 (12.7)
Fast foods are often considered healthier than home‐cooked or restaurant foods.	22 (7.3)

The adjusted Poisson regression model showed that students younger than 25 years consumed fast food 51.0% more frequently than those older than 25 years (incidence rate ratio [IRR] = 1.51, 95% confidence interval [CI] = 1.09–2.09, and *p* = 0.012). Additionally, with increasing education level, the frequency of fast food consumption decreased by 15% (IRR = 0.85, 95% CI = 0.75–0.97, and *p* = 0.018). No statistically significant associations were observed between BMI, gender, or marital status and fast food consumption frequency (Table 4).

**Table 4:** Factors related to the frequency of fast food consumption among students (*N* = 300).

Variables		Adjusted IRR^†^ (95% CI^‡^)^††^	*p* value
Gender	Male	1	—
	Female	1.04 (0.79–1.36)	0.773

Age, year	> 25	1	—
	≤ 25	1.51 (1.09–2.09)	0.012

Education	Continuous	0.85 (0.75–0.97)	0.018

Marital status	Married	1	—
	Single	0.86 (0.64–1.17)	0.349

Body mass index	Normal	1	
	Overweight and obese	1.10 (0.82–1.47)	0.528

^†^Incidence rate ratio.

^‡^Confidence interval.

^††^Adjusted for sex, age, education level, marital status, and body mass index.

## 4. Discussion

### 4.1. Prevalence and Trends

This cross‐sectional study conducted among medical sciences students in Kermanshah, Iran, revealed a prevalence of 55.7% for fast food consumption in the past week. This finding aligns with both national and international evidence indicating a rising trend of fast food intake among university students [14, 18]. Comparable prevalence rates have been reported in various Iranian regions, such as 60.5% in Kurdistan (2019) [22], 80.7% in Qom (2018) [17], 87% in Rafsanjan (2020) [15], and 55% in Shiraz (2020) [23]. These data collectively suggest that fast food consumption has become a common dietary habit among students, likely driven by factors such as time constraints and limited nutritional awareness.

### 4.2. Motivations for Consumption

The primary motivations for fast food consumption in our study were ease of access (60.0%), lack of time (44.7%), and taste (42.3%). These results are consistent with previous research emphasizing convenience and sensory appeal as key determinants in students’ food choices [2, 14, 23]. Socioeconomic factors, including income and urban residence, have also been implicated in increased fast food consumption [24]. Studies conducted in Saudi Arabia [26], Bangladesh [18], and Nepal [23] similarly highlight lack of cooking skills and affordability as important contributors. Despite its popularity, fast food is often nutrient‐poor, underscoring the need for policy interventions aimed at increasing availability of healthier food options within academic environments.

### 4.3. Association With BMI

Although approximately one‐third of fast food consumers in our sample were overweight or obese, no statistically significant association was found between fast food consumption and BMI. Similar findings have been reported in Pakistan [24] and Saudi Arabia [25]. This lack of association may stem from limitations of BMI as a health indicator, such as its inability to differentiate fat from muscle mass, or from biases inherent in self‐reported dietary and anthropometric data. Additionally, variation in portion sizes and types of fast food consumed could obscure any true relationships. Nonetheless, multiple studies [4, 14, 18, 25–27] have demonstrated a positive correlation between frequent fast food intake and higher BMI. For instance, Banik et al. reported a fourfold increase in obesity risk among fast food consumers [18]. These findings highlight the multifactorial nature of obesity, involving genetics, physical activity, and overall dietary patterns [28]. Therefore, although our study did not identify a direct link, public health strategies should continue to target fast food consumption as part of obesity prevention efforts.

### 4.4. Sociodemographic Determinants

Age was significantly associated with fast food intake: students aged 25 years or younger were 51% more likely to consume fast food than their older counterparts. This is consistent with findings from a 2023 Turkish study [4] and a 2022 systematic review [14], both reporting higher consumption rates among younger populations, potentially due to lower nutritional awareness and greater reliance on convenience foods.

No significant gender differences were detected in our study, aligning with results by Hatta et al. [26]. However, other studies have reported mixed results, with some finding higher consumption among males (10–11) and others among females [17]. Such inconsistencies may reflect cultural, behavioral, and health belief differences across populations. Despite the lack of statistical significance, the observed trends in our data suggest that subtle gender‐related differences may exist and merit further exploration to inform tailored interventions.

Marital status was not significantly associated with fast food consumption; however, single students showed a 0.86 times higher rate of intake compared to married students. This pattern supports prior findings from Iran [17] and the United States [29], potentially attributable to differences in cooking skills and time availability. While not statistically significant, this trend could indicate meaningful behavioral distinctions warranting additional study.

Interestingly, fast food consumption decreased by 15% with each advancing educational level, suggesting that increased academic exposure may enhance nutritional awareness. This finding aligns with previous Iranian research [17] but contrasts with studies from Malaysia [16], which may reflect differences in public health education. Even when not statistically significant, such trends provide valuable insight into student behavior and can guide future health promotion efforts.

### 4.5. Implications and Recommendations

The high prevalence of fast food consumption, particularly among younger, less educated, and single students, underscores the urgent need for targeted interventions. Universities should consider policies promoting access to affordable and nutritious meals, integrating nutrition education into curricula, and addressing behavioral and environmental factors influencing food choices. Future research should also investigate portion sizes, meal timing, and qualitative determinants of fast food consumption to better inform effective public health strategies.

### 4.6. Limitations

This study has several limitations that should be acknowledged. First, its cross‐sectional design restricts the ability to infer causality between fast food consumption and associated factors. Second, the potential for social desirability bias exists despite assurances of anonymity during data collection. Third, reliance on self‐reported dietary intake, weight, and height may introduce recall bias or intentional underreporting. Fourth, the study population was limited to students from Kermanshah University of Medical Sciences, restricting geographic and demographic diversity and thus limiting generalizability. Fifth, the small number of participants in some subgroups, such as the Faculty of Food Industry, may have reduced the statistical power of subgroup analyses. Future studies with larger samples from these faculties are recommended. Finally, demographic imbalances in gender and educational level within the sample may have further affected representativeness.

## 5. Conclusions

This study highlights a considerable level of fast food consumption among medical sciences students and identifies several personal and contextual factors influencing this behavior. Since medical students are expected to model healthy lifestyles, promoting balanced dietary habits is crucial to prevent future health problems. Educational interventions aimed at improving nutritional awareness, along with institutional efforts to increase access to healthy and affordable food options on campus, are strongly recommended. Future research should further examine barriers to healthy eating and food safety concerns among university students.

## Ethics Statement

The Ethics Committee of the Kermanshah University of Medical Sciences approved the study with the code IR.KUMS.REC.1398.819. Written informed consent was obtained from all the participants. All the experiment protocols for involving humans were in accordance with the guidelines of the national/international/institutional or Declaration of Helsinki. All methods were carried out in accordance with relevant guidelines and regulations. Furthermore, all experimental protocols were approved by the Ethics Committee of Kermanshah University of Medical Sciences.

## Consent

The authors have nothing to report.

## Disclosure

All the authors read and approved the version for submission.

## Conflicts of Interest

The authors declare no conflicts of interest.

## Author Contributions

Maryam Janatolmakan, Mahnaz Ghowsi, Ali Soroush, Shahab Rezaeian, and Alireza Khatony contributed to the study design. Maryam Janatolmakan and Mahnaz Ghowsi collected the data, and Shahab Rezaeian analyzed it. Maryam Janatolmakan, Mahnaz Ghowsi, Shahab Rezaeian, Ali Soroush, and Alireza Khatony wrote the final report and manuscript.

## Funding

The study was funded by the Kermanshah University of Medical Sciences (Grant no. 980724). The budget was spent on sampling.

## Data Availability

The data that support the findings of this study are available on request from the corresponding author. The data are not publicly available due to privacy or ethical restrictions.
